# Identification and virtual screening of novel salty peptides from hydrolysate of tilapia by-product by batch molecular docking

**DOI:** 10.3389/fnut.2023.1343209

**Published:** 2024-01-08

**Authors:** Hongjun Ren, Jingxuan Zhou, Huixian Fu, Qiaohui Feng, Jionghao Wang, Chuan Li, Guanghua Xia, Wenting Shang, Yanfu He

**Affiliations:** ^1^College of Food Science and Engineering, Hainan University, Haikou, China; ^2^Hainan Provincial Engineering Research Centre of Aquatic Resources Efficient Utilization in the South China Sea, Haikou, China; ^3^Key Laboratory of Seafood Processing of Haikou, Haikou, China

**Keywords:** tilapia by-product hydrolysate, salty peptide, salt receptor, TRPV1, virtual screening, molecular docking

## Abstract

**Introduction:**

Tilapia produces a large number of by-products during processing, which contain potentially flavorful peptides.

**Methods:**

The application of PyRx software enabled batch molecular docking andscreening of 16 potential salty peptides from 189 peptides identified in the enzymaticdigestion of tilapia by-products.

**Results:**

According to sensory analysis, all 16 peptides werepredominantly salty with a threshold of 0.256 - 0.379 mmol/L with some sournessand astringency, among which HLDDALR had the highest salty intensity, followedby VIEPLDIGDDKVR, FPGIPDHL, and DFKSPDDPSRH. I addition, moleculardocking results showed these four core peptides with high salt intensity bound to thesalt receptor TRPV1 mainly via van der Waals interactions, hydrogen bonds, andhydrophobic forces; Arg491, Tyr487, VAL441, and Asp708 were the key sites for thebinding of salty peptides to TRPV1. Therefore, the application of batch moleculardocking using PyRx is effective and economical for the virtual screening of saltypeptides.

## 1 Introduction

As standards of living improve, people are becoming increasingly concerned about the health of their diets. Many diseases such as hypertension, kidney disease, cardiovascular disease, and osteoporosis are associated with excessive salt intake, so there is a need to develop salt substitutes that reduce sodium intake while maintaining the saltiness of foods. Many researchers have proposed different solutions to reduce sodium chloride (NaCl) content in foods without altering the original salty taste, such as substitution with other metallic salts, the addition of flavor enhancers, and improvement of NaCl taste perception by taste contrast, and several salt substitutes have been reported to reduce dietary salt levels in foods; however, the successful implementation of salt reduction strategies depends on several factors, including their effects on food taste. Alio et al. (1) used KCl, CaCl_2_, and MgCl_2_ as partial substitutes for NaCl in dry-cured ham; better dehydration and curing were observed with KCl replacing 50% of NaCl, whereas CaCl_2_ and MgCl_2_ partially replacing NaCl resulted in a burnt taste quality defect. However, too high of a replacement with KCl can lead to bitter and metallic flavors, e.g., replacing 50% NaCl with KCl in bread making is organoleptically acceptable but leaves a poor aftertaste and flavor as the potassium salt itself may give a bitter/metallic off-flavor (2). Flavor enhancers, such as monosodium glutamate (MSG), inorganic phosphate nucleotides (IMP/GMP), herb and spice blends, certain alkaline amino acids, and salty peptides (3), are another important salt reduction strategy that can increase the perception of salt when mixed with salt, but not their salty taste. However, MSG and IMP/GMP still contain a certain amount of Na ions and carry some risk of harm to the body.

Although they have a salty taste, salty peptides do not contain Na ions, and their nutritional and functional characteristics have received widespread attention. They can replace salt for food cooking or processing to a certain extent. Their use, development, and application prospects are broad (3). Zheng et al. (4) discovered five salty peptides of ASP-Asp, Glu-ASP, ASP-Asp-Asp, Ser-PRO-Glu, and PHE-DILE from yeast extracts. Mushroom fungi are the main source of microbial salty peptides, and (5) isolated pyroglutamine dipeptide from mushroom protein hydrolysate, which can improve the salty taste of a solution. A polypeptide EDEGEQPRPF was separated and purified from commercial soy by Chen et al. (6), which not only has a salty taste, but also has a synergistic effect with NaCl, that is, the salty strength of 0.4 mg/mL salt peptide added to 50 mmol/L NaCl solution is equivalent to 63 mmol/L NaCl solution, and the salty flavor strength is increased by 26%.

Tilapia, scientifically known as *Oreochromis mossambicus*, is a tropical fish of African origin that is capable of living in highly saline waters. Numerous by-products containing potentially bioactive proteins are produced during the processing of tilapia, of which the rational exploitation provides abundant high-quality protein resources for humans, and the preparation of protein hydrolyzed flavor peptides using aquatic by-products has been a hot research topic.

Salinity receptors include both transient receptor potential cation channel subfamily V member 1 (TRPV1) and epithelial Na^+^ channels (ENaCs) (7, 8). ENaCs play a major role in Na^+^-triggered salt signaling and are highly sensitive to amiloride administration, whereas TRPV1 is a cation non-selective pathway that is insensitive to one or more amilorides (9). TRPV1 is best known as the capsaicin receptor, but can also be activated by calmodulin, peptides, endogenous lipids, ATP, acidity (pH <5.9), and temperature (>43°C), suggesting that it is a pleiotropic receptor (10). Investigation of the TRPV1 receptor structure revealed that its three-dimensional structure resembles the homotetramer of a voltage-gated ion channel (VGIC) with six highly conserved transmembrane (TM) structural domains (S1 ± S6), with transmembrane helices S5 and S6 forming the ion permeation pathway and transmembrane helices S1 to S4 forming the central pore ring region (11). Salty peptides can generate cations by ionization, some of which are used in taste transduction processes to convert chemical signals into molecular second messengers, causing the interaction between cations and taste receptor cells, activating ion channels on taste receptor cells’ surfaces, and triggering depolarization and Ca^2+^ release from taste receptor cells, which in turn transmits the “salty” signal to the brain (12).

The identification of flavor peptides from food constituents is an important challenge. Although traditional multiplex chromatographic purification methods can identify small amounts of peptides, they are time-consuming and may affect the accuracy of the results (13). Currently, gel filtration, ion exchange, and reversed-phase high-performance liquid chromatography (HPLC) are commonly used to determine peptide fractions in hydrolysis products or aqueous extracts, followed by tandem mass spectrometry for peptide identification (5, 6, 14). Therefore, the identification of salt peptides requires high throughput, high sensitivity, and high accuracy methods. Virtual screening is a class of screening techniques consisting of bioinformatics, molecular docking, and other techniques (15). Many studies have accurately screened peptides with different flavors using molecular docking techniques (16–19).

Consequently, in the present study, we propose to use tilapia by-products to prepare taste-active peptides by enzymatic digestion, identify salty taste peptides using a high-throughput screening method based on peptidomics and virtual screening, and further evaluate the binding mechanism of salty peptides to the TRPV1 receptor to lay the foundation for the application of developing salty substances of natural origin.

## 2 Materials and methods

### 2.1 Materials

Tilapia by-products were purchased from Hainan Xiangtai Fishery Co, Ltd. (Chengmai County, Hainan Province, China), boiled in water for 15 min to inactivate the endogenous protease activity, then stirred, portioned, and stored at −20°C. The potential salt peptides were synthesized by Nanjing Peptide Biotechnology Co. Ltd. (Nanjing, Jiangsu Province, China), with a purity of 98%. Papain was purchased from Henan Shengsted Industrial Company (Zhengzhou, Henan Province, China). All chemicals and solvents were analytical grades.

### 2.2 Preparation of salty peptide component by hydrolyzing tilapia by-product

A previous study by our group found that tilapia by-products hydrolyzed with papain had the highest salty taste score. Therefore, the preparation of enzymatic hydrolysate of tilapia by-products was based on the method of Gan et al. (20). By-products were added to distilled water at a solid–liquid ratio of 1:3 and homogenized in an ice bath. After being pH-adjusted with 1 mol/L NaOH or HCl solution, the mixture was shaken in a constant temperature and shaker water bath. Based on the results of our previous study, the optimal enzymatic conditions for papain are as follows: 0.8% enzyme addition, 50°C enzymatic temperature, pH 7.5, and 6 h enzymatic time. Enzymatic products were inactivated at 95°C for 15 min.

Enzyme hydrolysate was centrifuged and ultrafiltered at 4°C using an ultrafiltration tube (Millipore, Massachusetts, United States) with a cut-off relative molecular weight (MW) of 2.0 kDa. Fractions with a MW less than 2.0 kDa were retained for subsequent peptide identification.

### 2.3 Peptide identification

Liquid chromatography-tandem mass spectrometry (LC-MS/MS) detection: After desalting, each sample was separated by the nanoUPLC liquid phase system EASY-nLC1200 and data were collected using a mass spectrometer equipped with a nanoliter ion source. The mobile phase was an acetonitrile-water-formic acid system, where mobile phase A contained 2% acetonitrile, 98% H_2_O, and 0.1% formic acid, and phase B contained 80% acetonitrile, 20% H_2_O, and 0.1% formic acid. After equilibration of the column with 100% phase A, the sample was injected through an autosampler and separated by a column gradient at a flow rate of 300 nL/min and a gradient duration of 120 min. The mobile phase B ratio was: 2%–5% for 2 min, 5%–22% for 88 min, 22%–45% for 26 min, 45%–95% for 2 min, and 95% for 2 min. Mass spectrometry (MS) was performed in data-dependent acquisition mode with positive ion detection, 120k resolution, automatic gain control (AGC) of 3E6, max IT 50 ms, and a primary scan range of 350–1,600 *m*/*z*. The 20 most intense ions in the primary scan were screened by a four-stage rod and then scanned for fragment ions using high-energy induced cleavage with 15k resolution and an AGC of 1E5, max IT 50 ms. The dynamic exclusion time was set to 45 s according to the peak width; the secondary scan was not performed for single-charged and >6-valent ions.

Identification of library searches and protein quantification: raw data files were first converted to the common mzML file format using ProteoWizard software (version 3.0.18299). The MS data were matched to the corresponding species-level database sequence (uniprotOreochromis+Niloticus.fasta) using MSFragger software, and the main search parameters were according to the developer’s recommendations. Enzyme specificity was set to: non-specific; allowed peptide length range: 7–25; variable modifications included: 15.994915 [M], 42.010565 [nterm]; [C] without alkylation modification; parent ion mass accuracy: +/20 ppm; fragment ion accuracy: +/20 ppm. MSFragger library search results were then analyzed using the Philosopher (v3.3.11) toolset for subsequent analysis, primarily including PeptideProphet (v5.2.1) for peptide false discovery rate (1%) control and ProteinProphet (v5.2.1) for protein false discovery rate (1%) control. Quantification analysis was performed using the frequent module, and additional maxLFQ analysis was performed using IonQuant with a min ratio count of 1.

### 2.4 Homology modeling of salty taste receptor

The crystal structure of TRPV1 as a human salt receptor remains unclear, therefore, homology modeling was used to construct its three-dimensional (3D) structure. The amino acid sequence of TRPV1 was obtained from the UniProt database at https://www.UniProt.org/ (protein accession number Q8NER1). The template (PDB: 7LQY) was selected and homology modeling was performed using SwissModel.^1^ To further assess the quality of the model, the modeling was performed using SAVES,^2^ and the optimized homology model was evaluated using the residual percentage of Ramachandran plots (PROCHECK). The validated homology model was further used for molecular docking.

### 2.5 Virtual screening of salty peptides by molecular docking

Peptide structures were drawn using Chem Draw 14.0 (Cambridge soft, Cambridge, United States) and hydrogen atoms were added to the CHARMM force field in PyMOL (Schrödinger, LLC., New York, NY, United States). Batch energy minimization and format conversion of 3D peptide structures using PyRx (SourceForge, San Diego, United States) and batch molecular docking of 3D peptide structures to receptors accelerated the virtual peptide screening process and reduced operational complexity (21). The AutoDock Vina docking software (Scripps Research Institute Molecular Graphics Laboratory, La Jolla, CA, United States) included with PyRx was used to perform molecular docking. A semi-flexible docking method was chosen for the docking process. Peptides with docking energy < −8 kcal/mol were further synthesized and verified.

### 2.6 Validation of salty peptides

#### 2.6.1 Sensory evaluation

Sensory evaluation was based on the method proposed by Gan et al. (20) with some modifications. The sensory evaluation team consisted of eight sensory evaluators, aged 20–25 years, four males and four females, who were uniformly screened and professionally trained. The evaluators were trained in advance using 50 mg/mL NaCl as a reference solution for salty taste. The salty taste of each synthetic peptide was rated at room temperature (25 ± 1°C) using the 10-point method, with 0–3 being weak, 4–6 being standard, and 7–10 being strong.

#### 2.6.2 Taste dilution analysis

To further understand the flavor properties, the taste dilution analysis (TDA) method was used to assess the flavor recognition thresholds of the synthetic peptides and to define their palatability. Taste peptides were prepared at a concentration of 1 mg/mL and diluted in a 1:1 series with deionized water until just separated from the blank control. The maximum dilution concentration that could be accurately judged by trained sensory panel members was used as the salty taste detection threshold for the peptide.

#### 2.6.3 Electronic tongue analysis

According to the method of Zhang et al. (15), the peptides were prepared at a concentration of 0.5 mg/mL, loaded into special beakers for the electronic tongue, and placed on an autosampler and analyzer. Each sample was repeated four times and the data from the first time were removed. The response characteristics of the Insent SA402B electronic tongue sensors AAE, CT0, CA0, C00, and AE1 were fresh, salty, sour, bitter, and astringent flavors. The sensor and reference probes were immersed in the sample solution for 30 s to detect membrane potential changes and the same concentration of KCl solution was used as a standard control.

### 2.7 Statistical analysis

SPSS 23.0 was used to process the relevant data for ANOVA and significance testing, and each group of experiments was repeated three times to calculate the mean; Origin 2022 was used for graphing.

## 3 Results and discussion

### 3.1 Identification of salty peptides from hydrolysate of tilapia by-product by nano-HPLC-MS/MS

Previous studies found that the MW of peptides with high flavor activity was mainly concentrated in the range of <1,500 Da (22). Therefore, in this study, hydrolysate with a strong salty flavor was ultrafiltered and the AA sequences of peptides in the ultrafiltered fractions were determined by nano-UPLC-MS/MS.

A total of 189 peptides (Supplementary Table S1) were detected in three parallel samples, 85.6% of which had molecular weights <1,500 Da, and the sequence length distribution of the identified peptides was analyzed (Supplementary Figure S1), which mainly consisted of 12–14 AAs, followed by 10–11 AAs, with a smaller distribution of peptides with less than 10 AAs. Subsequently, 189 peptide sequences were used for the virtual screening of potential salty peptides.

### 3.2 Homology modeling of TRPV1 receptor

**Figure 1:**
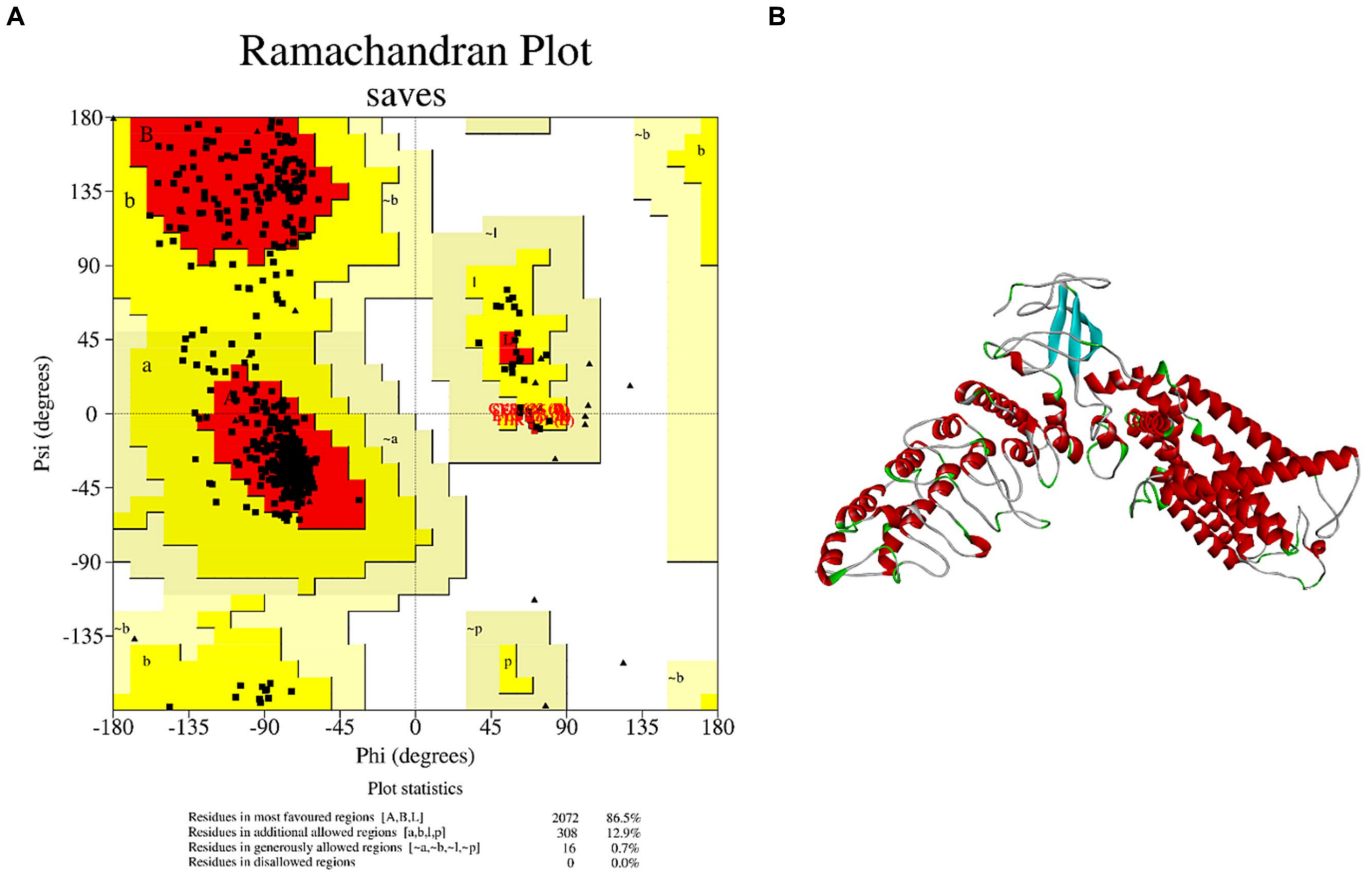
Homology modeling of TRPV1 and its reliability. **(A)** Ramachandran plot of TRPV1. **(B)** 3D structure of salty receptor TRPV1 subunit.

Although TRPV1 is associated with salty taste sensitivity (23), few studies have elucidated the mechanism of salty taste by molecular docking between salty taste receptor TRPV1 and salty substances. There is currently no definitive 3D structure of TRPV1 in the Protein Structure Data Bank (PDB); therefore, a model of the TRPV1 receptor must be obtained via homology modeling. The AA sequence of TRPV1 was searched in the Protein Data Bank (Uniprot) and the homologous sequence obtained, Q8NER1, was used as a template for TRPV1 to be submitted to the SwissModel server for homology modeling analysis. TRPV1 with PDB ID 7LQY was selected as the template to construct the best model. The analysis results showed that the sequence similarity of TRPV1 was 85.35%. Meanwhile, the Ramachandran plot calculated by SVAE showed that 100% of the AA residues were within a reasonable range, where the optimal region was 86.5%, the acceptable region was 12.9%, the generally acceptable region was 0.7%, the disagreed region was 0.00% (Figure 1A). It is generally accepted that the sequence similarity between the target and template is 30% (24) and the AA residues in the disallowed region are <5% (25). Thus, the homologous model was successfully used to predict the protein structure. Since TRPV1 is a homotetramer protein consisting of four monomers, the A chain of TRPV1 was extracted as an acceptor (TRPV1 A) for molecular docking (Figure 1B). These results suggest that the TRPV1 model constructed in this study can be used as a receptor for further docking analysis.

### 3.3 Virtual screening of potential salty peptides by molecular docking and their taste characteristics

**Figure 2:**
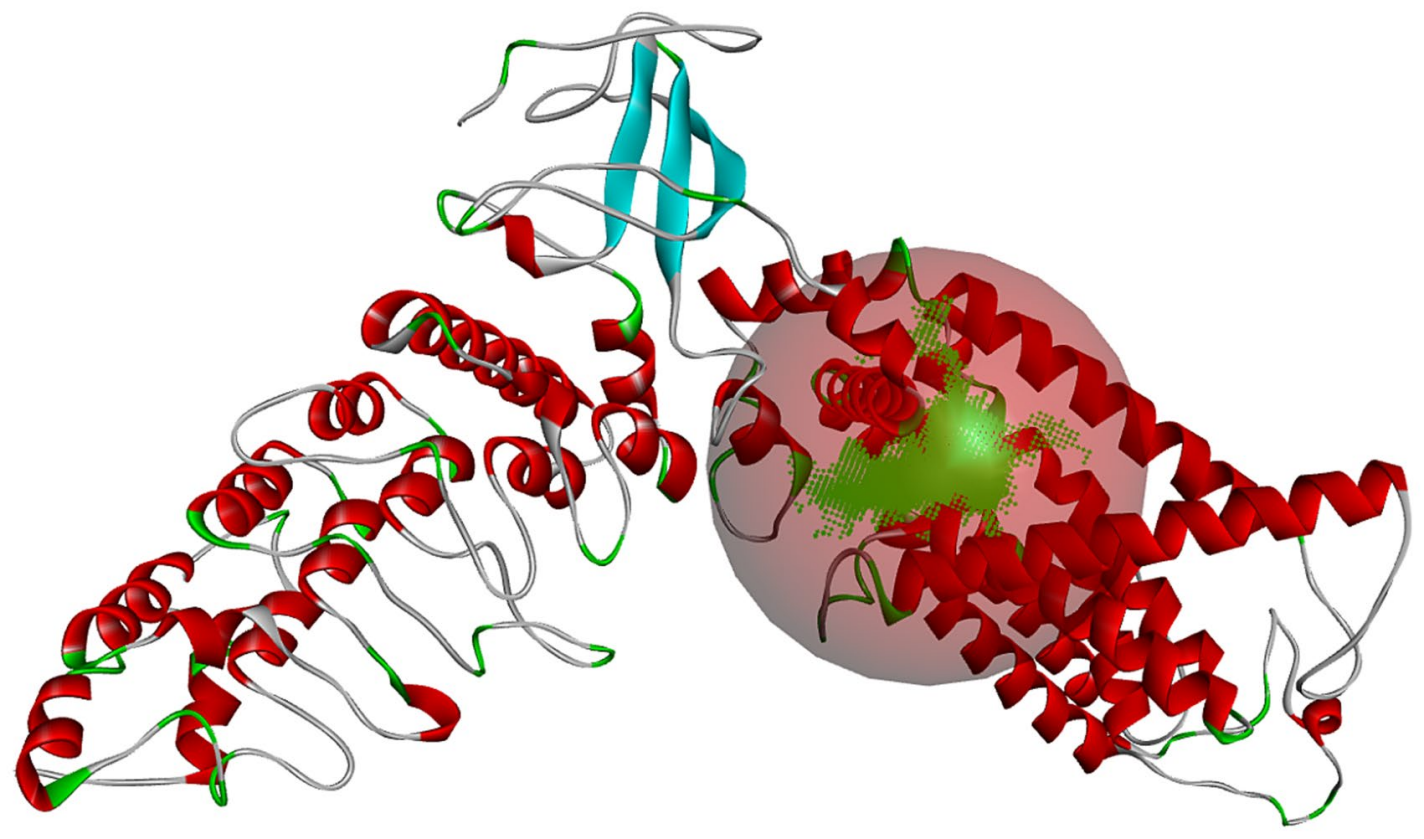
The predicted binding pocket of salty receptor TRPV1. The green area and red balls form the active binding pockets of the salty receptor TRPV1.

After establishing a reasonable homology model, the binding pocket of the salty receptor TRPV1 was predicted by Discovery Studio as shown in Figure 2, where the predicted pocket is composed of red spheres and the green represents the binding sites in the binding pocket. Similar to the binding pocket of TRPV1 as a capsaicin or cannabinoid receptor (19, 26), the binding pocket is formed by S3, S4, and S4–S5 junctions in the membrane (27). However Li et al. (26), found that endogenous cannabinoids can also bind to TRPVl, but the details of the ligand-TRPVl interaction are different from those of capsaicinoids; hydrogen bonds between capsaicinoids and T551 are important for their binding, but no hydrogen bonds are formed between endogenous cannabinoids and T551. They suggested that van der Waals interactions between the tail of the ligand and the receptor channel protein are the main factors affecting the binding conformation of endogenous cannabinoids to TRPVl. Thus, differences in the binding pocket in terms of key AA residues, ligand structure, and the forces formed by the AA residues with the ligand may be important for the different tastes perceived by TRPV1 as a receptor. The binding pocket was, therefore, used to further investigate the binding mechanism between the salty peptide and TRPV1 and red balls form the active binding pockets of the salty receptor TRPV1.

Batch molecular docking of the ligand to the TRPV1 receptor was performed by generating 189 peptide 3D structure files and using AutoDockVina under PyRx. The docking energies of the 189 peptides are listed in Supplementary Table S1. Docking energy is the main reference indicator for screening ligand-receptor interactions, and the value of the docking energy indicates the strength of affinity between the ligand and receptor. Sixteen of 189 peptides were screened according to the docking energy, as shown in Supplementary Table S2, and their docking energies are less than −8 kcal/mol; docking energy less than −7.0 kcal/mol indicates a strong binding activity between ligand and receptor, and the lower the docking energy, the more stable the receptor-ligand complex (28). As more than 60 of the 189 peptides had docking energies less than −7 kcal/mol (Supplementary Table S1), we selected 16 peptides with docking energies less than −8 kcal/mol for further analysis.

To further understand the taste properties of the salty peptides, the taste recognition thresholds of 16 synthetic peptides were evaluated using TDA, and their taste ability was defined. As shown in Supplementary Table S2, the taste recognition thresholds of the 16 peptides ranged from 0.256–0.379 mmol/L, and their salty taste thresholds were all lower than that of salt, indicating that the synthetic peptides were highly salty, but many of them also had some sour and astringent tastes. Chen et al. (6) identified the taste active peptides from Chinese curd; Glu-Asp-Glu-Gly-Glu-Gln-Pro-Arg-Pro-Phe was the most promising salt-tasting peptide and a 50 mmol/L NaCl solution containing 0.4 mg/mL of the peptide was equivalent to the salt-tasting level of a 63 mmol/L NaCl solution. Shan et al. (29) reported that four peptides, PKLLLLPKP, GGISTGNLN, LVKGGLIP, and SSAVK isolated from yeast extract had high salt-taste enhancing effects with their thresholds ranging from 0.18–0.59 mmol/L.

**Figure 3:**
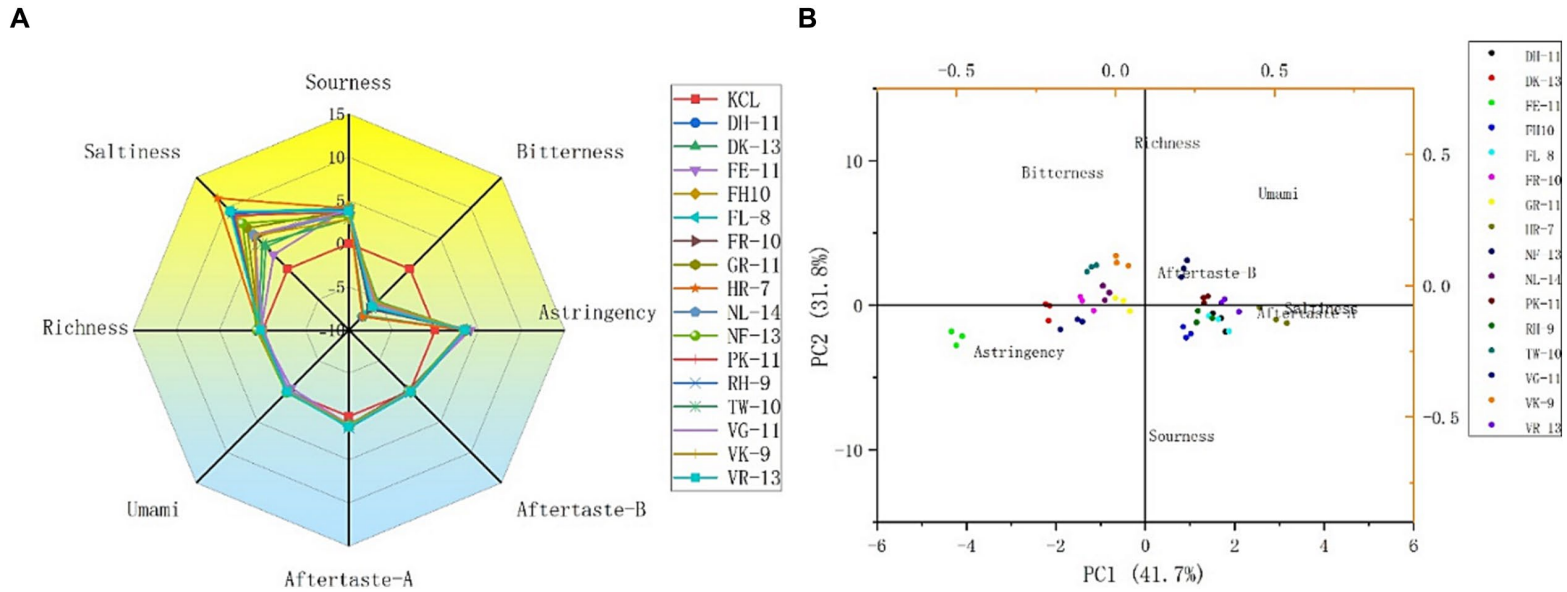
Taste radar map **(A)** and principal component analysis (PCA) plot **(B)** of the 16 potential salty peptides analyzed by e-tongue.

The 16 potential salty peptides identified from the enzymatic digestion of tilapia by-products were synthesized and their salty taste characteristics were verified using the electronic tongue system. The principal component analysis (PCA) and taste radar plots of the 16 synthesized peptides by e-tongue analysis are shown in Figures 3A,B. The taste characteristics of the 16 synthetic peptides had the same trend, where salty was the strongest, followed by sour and astringent; moreover, the 16 peptides had the same intensity of astringency, fresh taste, and richness, bitter aftertaste (aftertaste-A) and astringent aftertaste (aftertaste-B) were not prominent, and bitter was the weakest, with their intensities lower than that of KCl (Figure 3A). Among the 16 synthetic peptides, HLDDALR had the highest salty taste intensity score (11.56), followed by VIEPLDIGDDKVR (9.47), FPGIPDHL (9.30), and DFKSPDDPSRH (9.17). However, as shown in Supplementary Table S2, the peptides with the lowest docking energies were FPGDFTPEVH and VIEPLDIGDDKVR, both at −9 (kcal/mol), followed by GEIDEFLPAPR and VFDISNADRLG, both at −8.6 (kcal/mol). The salt intensity of several of the 16 peptides, except VIEPLDIGDDKVR, was not particularly prominent, suggesting that the docking energies were not consistent with the salt intensity. Therefore, we speculated that although molecular docking can be used to predict the taste characteristics of salty peptides, the intensity of salty taste cannot be predicted by the docking energy alone and this must be combined with sensory evaluation and e-tongue. This is similar to the results of previous studies (30, 31).

To further analyze the differences between the different peptides, the e-tongue response scores of freshness, saltiness, sourness, bitterness, bitter aftertaste, and astringent aftertaste in the samples were selected for PCA, and the contributions of PC1 and PC2 were 41.7% and 31.8%, respectively (Figure 3B). Salty aftertaste had the greatest effect on PC1, followed by astringent and bitter aftertaste, while sourness and richness had a greater effect on PC2. Sixteen peptides were separated mainly along PC1 and four peptides, HLDDALR, VIEPLDIGDDKVR, FPGIPDHL, and DFKSPDDPSRH, were similarly distributed and close to the salty taste. It was further confirmed that the four salty peptides had a relatively high salty taste intensity.

### 3.4 Energy interaction and surface force between TRPV1 receptor and salty peptides

**Figure 4:**
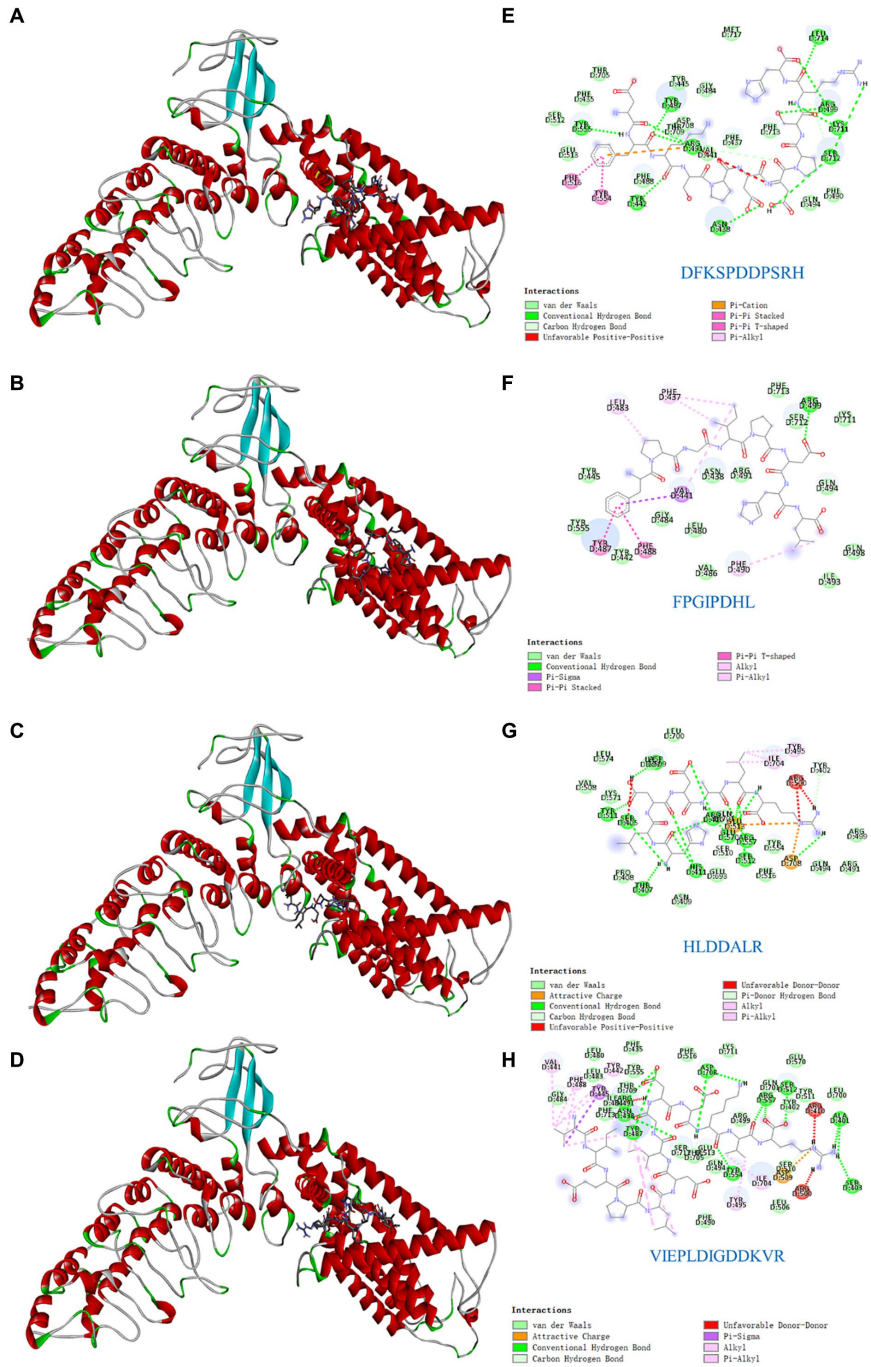
Molecular docking of the TRPV1 receptor and salty peptides. **(A–D)** 3D docking complex diagrams between TRPV1 and four salty peptides. **(E–H)** 2D diagrams between TRPV1 and four salty peptides.

Understanding the key active sites of flavor peptides is important for understanding ligand and receptor recognition patterns (15). To explore the mechanism of taste presentation of salty peptides, we further investigated the interaction between TRPV1 and four salty peptides. Molecular docking is a process in which two or more molecules recognize each other through assembly and energy matching to obtain the optimal binding pattern between the molecules (32). The optimal binding conformation of the four salty peptides in the active pocket of TRPV1 is shown in Figures 4A–D and the 2D plot of the interaction between the four peptides and the active residues of TRPV1 is presented in Figures 4E–H. The results showed that the four salty peptides were successfully inserted into the active site of the TRPV1 subunit and were locked in the active pocket of TRPV1.

The binding forces of ligand-receptor interactions in the spatial structure have a strong influence on taste, including electrostatic interactions, hydrogen bonding, hydrophobic interactions, π-alkyl, π-sulfur, salt bridges, and van der Waals force interactions (15). As shown in Figures 4E–H, the binding of the four salty peptides to TRPV1 mainly involves non-covalent interactions, such as van der Waals interactions, hydrogen bonding, and hydrophobic forces (alkyl or π-alkyl). In addition to van der Waals forces, the conventional hydrogen bond and alkyl or π-alkyl are the main interaction forces between the four salty peptides and TRPV1, while π-stacked is only present in DFKSPDDPSRH and FPGIPDHL, and the attractive charge is only present in HLDDALR and VIEPLDIGDDKVR. The most bindings between VIEPLDIGDDKVR and TRPV1 included 13 alkyl or π-alkyl and 12 conventional hydrogen bonds, two π-stacked, and the rest were two unfavorable donor–donor, one attractive charge, and one π-sigma. π-alkyl is a hydrophobic force. Hydrophobic interactions, as well as the balance between hydrophobic and hydrophilic forces, are the main forces that maintain structural stability between peptides and receptors (30); hydrogen bonding also plays an important role in biological macromolecules, being one of the main driving forces for protein-ligand interactions and playing an indispensable role in stabilizing protein-ligand complexes. Conventional hydrogen bonds were also as high as 12 and 15 in DFKSPDDPSRH and HLDDALR, respectively, while there was only one conventional hydrogen bond in FPGIPDHL. However, another five alkyl or π-alkyl interactions, two π-π stacked and one π-Sigma interaction, in which residue VAL441 formed both π-π stacked and π-Sigma interactions with FPGIPDHL, made us speculate that VAL441 may be a key AA for the binding of FPGIPDHL to the salty receptor TRPV1 residues. It is possible that the differences in molecular structure led to the differences in receptor-ligand interactions, which ultimately led to the differences in the affinity of the peptides for TRPV1, which is similar to the findings of Xiao et al. (19), and these strong interactions may lead to a better salty taste of the salty peptide.

In this study, four salty peptides exhibited binding to 37 AA residues of the TRPV1 subunit, the major AA residues being Arg491, Tyr487, VAL441, Asp708, Arg557, Asp509, Arg410, Arg499, Arg500, Tyr445, and Phe488, with Arg491 appearing frequently in DFKSPDDPSRH and VIEPLDIGDDKVR; Tyr487 and VAl441 occurred in DFKSPDDPSRH, FPGIPDHL, and VIEPLDIGDDKVR; and Asp708 occurred in HLDDALR and VIEPLDIGDDKVR. These results suggest that Arg491, Tyr487, VAL441, and Asp708 are important binding sites that play a crucial role in the interaction between salty peptides and TRPV1 subunits. TRPV1 has been extensively studied as a capsaicin receptor, and Domene et al. (33) reported that the binding of TRPV1 to capsaicin is mediated by hydrogen bonding and van der Waals interactions and that residue Y511 is essential for stabilizing the binding state and during the binding process. Xiao et al. (19) reported that some amide bonds and similar groups, or even benzene rings in spicy compounds, play a key role in spicy taste perception, with Glu570 in the active pocket of TRPV1 playing an important role in spice detection. There are similarities but also differences in the binding modes of TRPV1 and ligands as receptors for different tastes and perceptions. The similarities lie in the binding modes of different ligands to TRPV1 being mainly hydrogen bonds and van der Waals forces, while the differences lie in the different binding AA residues.

**Figure 5:**
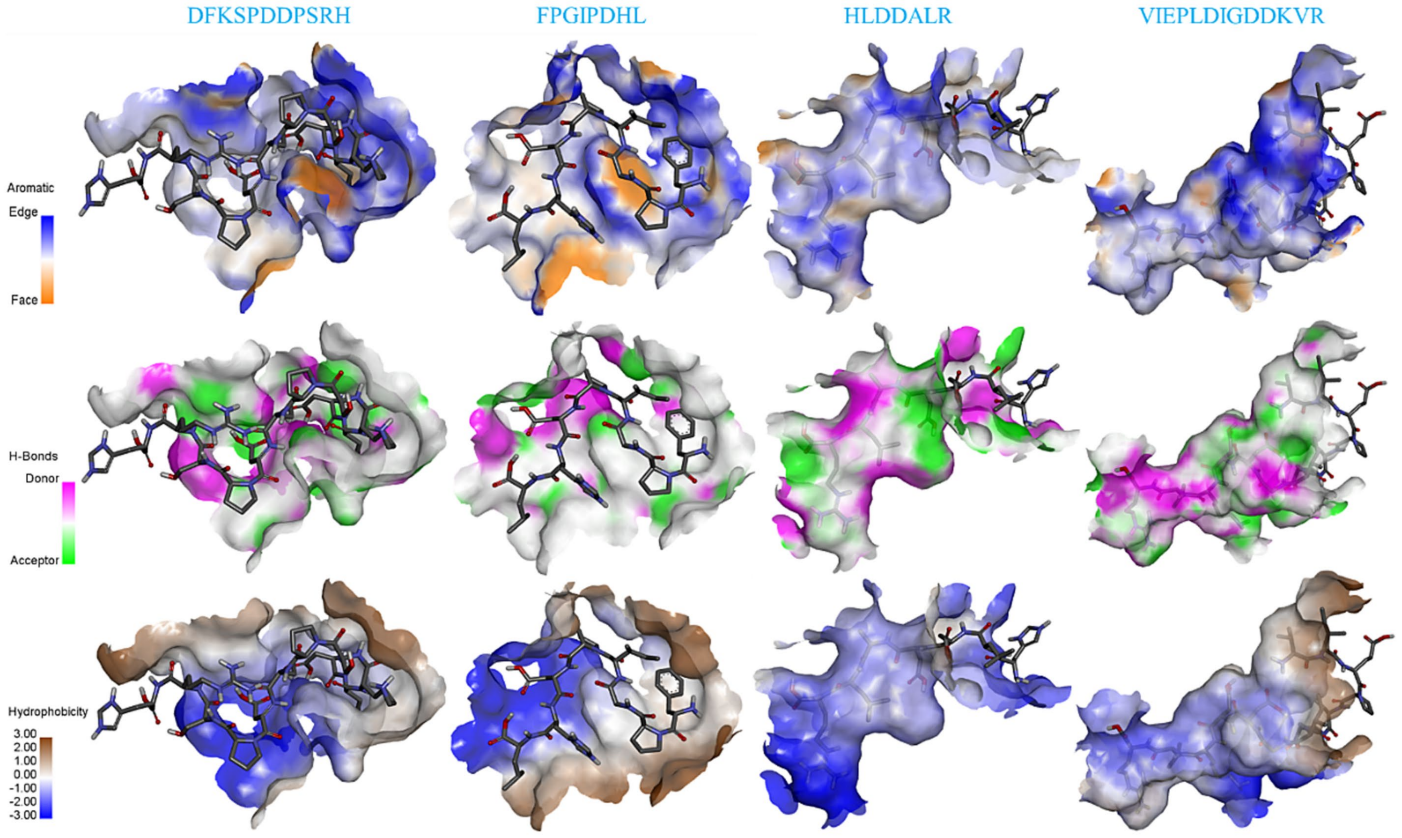
Surface force analysis of TRPV1 interaction with four salty peptides.

Figure 5 shows the details of the surface forces between the salty peptides and TRPV1. The HLDDALR and VIEPLAIGDDKVR structures are mostly wrapped in the lumen of the receptor TRPV1 active pocket, while DFKSPDDPSRH and FPGIPDHL are located on the surface of the receptor TRPV1 active pocket. All four salty peptides interact with TRPV1 through three main surface forces: aromatic interactions, hydrogen bonding, and hydrophobicity. In this study, the electrostatic interaction between the protons on the edge ring of TRPV1 and the salty peptide was stronger than the π-electron density on the face ring; this result is similar to the surface interaction between umami peptides and umami receptor T1R1/T1R3 (34). All four salty peptides were able to form strong hydrogen bonds with TRPV1; in terms of hydrophobicity, all four salty peptides were significantly hydrophilic, which may be attributed to the abundance of hydrophilic groups such as –NH_2_, –COOH, and –OH in TRPV1 and the salty peptide.

## 4 Conclusion

In this study, the major salty components of the enzymatic hydrolysate of tilapia by-products were obtained and identified to 189 peptides by nano-HPLC-MS/MS. Batch molecular docking of 189 peptides with the salty receptor TRPV1 was performed using Python script to obtain 16 potential salty peptides with low docking energy, which were further synthesized and subjected to taste identification and threshold analysis by sensory evaluation, TDA, and e-tongue. It was found that all 16 potential salty peptides had a significant salty taste with salty threshold values in the range of 0.256–0.379 mmol/L, which was lower than the salty threshold of NaCl. The highest salinity score was obtained for HLDDALR, followed by VIEPLDIGDDKVR, FPGIPDHL, and DFKSPDDPSRH. Further analysis of the binding forces and binding sites of these four salty peptides to TRPV1 revealed that van der Waals interactions, hydrogen bonding, and hydrophobic forces were the main binding forces. Aromatic interactions, hydrogen bonding, and hydrophobicity were the main surface forces and Arg491, Tyr487, VAL441, and Asp708 were the key sites for binding the salty peptides to TRPV1.

In summary, our work accomplished the preparation of enzymatic digests of tilapia by-products, virtual screening of salty peptides, and receptor binding analysis, which may provide a valuable research basis for the utilization of salty peptides from natural sources as well as salt reduction strategies.

The mass spectrometry proteomics data have been deposited to the ProteomeXchange Consortium^3^ via the iProX partner repository (35, 36) with the dataset identifier PXD047590.

## Data availability statement

The original contributions presented in the study are publicly available. This data can be found here: http://proteomecentral.proteomexchange.org, PXD047590.

## Author contributions

HR: Data curation, Investigation, Software, Writing – original draft. JZ: Investigation, Methodology, Software, Writing – review & editing. HF: Investigation, Methodology, Software, Writing – review & editing. QF: Conceptualization, Methodology, Writing – review & editing. JW: Methodology, Software, Writing – review & editing. CL: Resources, Visualization, Writing – review & editing. GX: Resources, Writing – review & editing. WS: Conceptualization, Resources, Writing – review & editing. YH: Formal analysis, Supervision, Writing – review & editing.

## Footnotes

^1^ https://swissmodel.expasy.org/

^2^ https://SAVES.mbi.UCLA.edu/

^3^ http://proteomecentral.proteomexchange.org
