# Supplementary material for: Identification and virtual screening of novel salty peptides from hydrolysate of tilapia by-product by batch molecular docking
Source: Front Nutr. 2024 Jan 8;10:1343209. doi: 10.3389/fnut.2023.1343209 (PMC10800615; doi:10.3389/fnut.2023.1343209)
Supplement: Supplementary file 1 [file Table_1.docx]

**Supplementary Table 1** 189 peptides identified from hydrolysate of tilapia by-product and their docking energy with TRPV1.

| Peptide Sequence | Sequence Length | Molecular weight | Protein ID | Protein Description | Docking Energy |
| --- | --- | --- | --- | --- | --- |
| AEKDEEMEQIK | 11 | 1349.46 | A0A669CK87 | Uncharacterized protein | -5.6 |
| AEVDNLSDAEER | 12 | 1347.34 | A0A669CK87 | Uncharacterized protein | -7.1 |
| AFTIIDQNRDGIIS | 14 | 1562.72 | A0A669BQ46 | Myosin light chain, phosphorylatable, fast skeletal muscle a | -5.9 |
| AGDSDGDGKIG | 11 | 990.97 | I3KV11 | Uncharacterized protein | -5.9 |
| AGDSDGDGKIGVD | 13 | 1205.19 | I3KV10 | Uncharacterized protein | -7.8 |
| AGQEDENGSVH | 11 | 1142.09 | I3K6V3 | Uncharacterized protein | -7.6 |
| AGQEDENGSVHYE | 13 | 1434.38 | I3K6V3 | Uncharacterized protein | -6.4 |
| AGRDLTDYLMK | 11 | 1282.47 | I3IYR2 | Uncharacterized protein | -6.2 |
| ARIEELEEELEAE | 13 | 1559.63 | A0A669CK87 | Uncharacterized protein | -6.9 |
| AVKNIDDLTGHL | 12 | 1295.44 | A0A669CKH1 | GLOBIN domain-containing protein | -6.7 |
| DAGTFDHKKFF | 11 | 1312.43 | A0A669EKH3 | Uncharacterized protein | -7.7 |
| DFDQFLPIHQ | 10 | 1259.37 | I3JSP5 | Uncharacterized protein | -7.1 |
| DFKSPDDPSRH | 11 | 1300.33 | A0A669C1U1 | 2-phospho-D-glycerate hydro-lyase | -8 |
| DKSGFIEEDELK | 12 | 1409.49 | I3KV11 | Uncharacterized protein | -5.8 |
| DLAGRDLTDYLM | 12 | 1382.54 | I3IYR2 | Uncharacterized protein | -7.1 |
| DMFEQIEENESL | 12 | 1483.55 | A0A669BVA8 | Uncharacterized protein | -5.5 |
| DMFEQIEENESLK | 13 | 1611.73 | A0A669BVA8 | Uncharacterized protein | -6 |
| DMPVGPILF | 9 | 988.2 | I3J5Q8 | Fibronectin | -7.9 |
| DPFDQDDWEAWTK | 13 | 1652.67 | A0A669C5U3 | 2-phospho-D-glycerate hydro-lyase | -8.1 |
| DPFDQDDWEH | 10 | 1303.25 | A0A669C1U1 | 2-phospho-D-glycerate hydro-lyase | -7.9 |
| DPFDQDDWEHWA | 12 | 1489.46 | A0A669C1U1 | 2-phospho-D-glycerate hydro-lyase | -7.1 |
| DPFDQDDWEHWAK | 13 | 1489.46 | A0A669C1U1 | 2-phospho-D-glycerate hydro-lyase | -7.1 |
| DPFNIHDLE | 9 | 1099.15 | A0A669BVQ8 | ATP-dependent 6-phosphofructokinase | -6.8 |
| DSFDYKKFF | 9 | 1196.31 | A0A669EX46 | Uncharacterized protein | -6.4 |
| DSFDYKKFFK | 10 | 1324.48 | A0A669EX46 | Uncharacterized protein | -6.5 |
| DTIDNDFKELH | 11 | 134640 | A0A669C9W7 | Uncharacterized protein | -6.5 |
| DVILPVPAF | 9 | 970.16 | A0A669C1U1 | 2-phospho-D-glycerate hydro-lyase | -6.6 |
| EDDIHPRNPPKFD | 13 | 1579.67 | A0A669CK87 | Uncharacterized protein | -6.2 |
| EDKSFTEVFVK | 11 | 1328.47 | I3KVU6 | Uncharacterized protein | -6.4 |
| EELDHALNDMTSL | 13 | 1487.59 | A0A669EEE4 | Uncharacterized protein | -6.1 |
| EFTPDQIEDFK | 11 | 1368.44 | I3K6V3 | Uncharacterized protein | -7.4 |
| EIWEDDVDGEHIIA | 14 | 1456.47 | A0A669D249 | Calsequestrin | -5.7 |
| EKDEEMEQIK | 10 | 1278.39 | A0A669CK87 | Uncharacterized protein | -4.3 |
| EKLHVDPDNFRVL | 13 | 1468.61 | A0A669EM02 | GLOBIN domain-containing protein | -7.5 |
| ELDANLKPIKPM | 12 | 1368.64 | I3J820 | Phosphoglycerate mutase | -5.7 |
| ELGEQIDNLQRVK | 13 | 1541.7 | A0A669CK87 | Uncharacterized protein | -6.7 |
| EMASQDETIAK | 11 | 1222.32 | A0A669BWI2 | Uncharacterized protein | -6.4 |
| ENKNLQEEISDLTE | 14 | 1431.5 | A0A669AZZ3 | Uncharacterized protein | -6.4 |
| EPEILPDGDHDLK | 13 | 1477.57 | I3KW15 | Fructose-bisphosphate aldolase | -7.5 |
| EPEILPDGDHDLKR | 14 | 1477.57 | I3KW15 | Fructose-bisphosphate aldolase | -6.2 |
| EPLDIGDDKVR | 11 | 1256.36 | A0A669BIT2 | Collagen type VI alpha 3 chain | -6.3 |
| EQFEEEQEAKAE | 12 | 1466.46 | A0A669CK87 | Uncharacterized protein | -5.9 |
| ERLEDEEEINAE | 12 | 1475.47 | A0A669CK87 | Uncharacterized protein | -4.5 |
| ERLEDEEEMN | 10 | 1293.31 | A0A669BWI2 | Uncharacterized protein | -4.9 |
| ERLEDEEEMNAE | 12 | 1493.51 | A0A669BWI2 | Uncharacterized protein | -5.8 |
| ESLMDLENDKQQ | 12 | 1449.54 | A0A669CK87 | Uncharacterized protein | -4.9 |
| ETFDEMIDEDEVK | 13 | 1599.67 | A0A493QWZ6 | Complement factor B | -6.2 |
| ETIMDLENDKQQ | 12 | 1463.57 | A0A669BWI2 | Uncharacterized protein | -5.4 |
| EVFPGIPDHL | 10 | 1123.26 | A0A669C9W7 | Uncharacterized protein | -7.8 |
| FEDNPDDHDRM | 11 | 1390.39 | A0A669C9W7 | Uncharacterized protein | -7.3 |
| FFVTDPDEMYVK | 12 | 1490.67 | A0A669BU68 | Uncharacterized protein | -7.4 |
| FFVVVPEEMYVK | 12 | 1486.77 | A0A669CK87 | Uncharacterized protein | -5.8 |
| FIEEDELKLF | 10 | 1282.44 | I3KV11 | Uncharacterized protein | -6.2 |
| FIYDVLEDPVE | 11 | 1338.46 | A0A669D249 | Calsequestrin | -8.2 |
| FPGDFTPEVH | 10 | 1145.22 | A0A669CKH1 | GLOBIN domain-containing protein | -9 |
| FPGIPDHL | 8 | 895.01 | A0A669C9W7 | Uncharacterized protein | -8.3 |
| FSADQIEDYR | 10 | 1243.28 | I3J8X4 | Uncharacterized protein | -8.5 |
| FSADQIEDYREAFG | 14 | 1443.47 | I3J8X4 | Uncharacterized protein | -6.6 |
| FSPKQPDLFK | 10 | 1206.39 | I3JBN0 | Alpha-1,4 glucan phosphorylase | -6.9 |
| FSVDDEFPDLTK | 12 | 1412.5 | I3JLI3 | Creatine kinase | -7.8 |
| FTAEEKLSIYK | 11 | 1328.51 | A0A669CK87 | Uncharacterized protein | -6 |
| FTIIDQNRDGIIS | 13 | 1491.64 | A0A669BQ46 | Myosin light chain, phosphorylatable, fast skeletal muscle a | -6.4 |
| FTPEQIEDFKDAFQ | 14 | 1439.52 | I3JSP5 | Uncharacterized protein | -6.8 |
| GEIDEFLPAPR | 11 | 1243.37 | A0A669F6M2 | Myomesin 1b | -8.6 |
| GEKMNEQEIDALM | 13 | 1376.49 | I3K6V3 | Uncharacterized protein | -5.8 |
| GEKVDFDDIQK | 11 | 1293.38 | I3JXH8 | Troponin T type 3a (skeletal, fast) | -6.5 |
| GFAGDDAPR | 9 | 904.92 | I3IYR2 | Uncharacterized protein | -6.6 |
| GMYFPGDFTPEVH | 13 | 1496.64 | A0A669CKH1 | GLOBIN domain-containing protein | -7.3 |
| GNEYIFRVM | 9 | 1128.3 | A0A669ETX1 | Uncharacterized protein | -6.3 |
| GNRDDEYDFLFK | 12 | 1518.58 | A0A669D830 | Uncharacterized protein | -6 |
| GPPVPGPIGPM | 11 | 1018.23 | I3JMB4 | Uncharacterized protein | -7.5 |
| GPPVPGPIGPMG | 12 | 1075.28 | I3JMB4 | Uncharacterized protein | -7.7 |
| GPPVPGPIGPMGPR | 14 | 1328.58 | I3JMB4 | Uncharacterized protein | -6 |
| GQEDENGSVH | 10 | 1071.01 | I3K6V3 | Uncharacterized protein | -7.8 |
| GSLEQEKKIR | 10 | 1187.35 | A0A669CK87 | Uncharacterized protein | -5.9 |
| GTLEHEEAKILR | 12 | 1395.56 | A0A669CK87 | Uncharacterized protein | -6.8 |
| GVFDISNADRLG | 12 | 1263.36 | I3JLI3 | Creatine kinase | -5.2 |
| GWLDKNKDPLN | 11 | 1299.43 | A0A669CK87 | Uncharacterized protein | -6.6 |
| HDLDQLLR | 8 | 1009.12 | I3JVB3 | Fibrinogen C-terminal domain-containing protein | -6.4 |
| HEKYDNSLKVVS | 12 | 1418.55 | A0A669BWU8 | Glyceraldehyde-3-phosphate dehydrogenase | -6.5 |
| HFEDNPDDHDRMF | 13 | 1674.7 | A0A669C9W7 | Uncharacterized protein | -6.2 |
| HLDDALR | 7 | 838.91 | A0A669CK87 | Uncharacterized protein | -8.4 |
| HPKFEEILTR | 10 | 1269.45 | I3JLI3 | Creatine kinase | -7.7 |
| HVDPDNFRVL | 10 | 1211.33 | A0A669EM02 | GLOBIN domain-containing protein | -7.1 |
| HYLDLKDMPFYA | 12 | 1441.65 | I3JS34 | Nucleoside diphosphate kinase B | -6.8 |
| IDHLNEEKLK | 10 | 1238.39 | I3JXH8 | Troponin T type 3a (skeletal, fast) | -4.7 |
| IDKIDEERYDLQ | 12 | 1536.64 | I3JXF9 | Uncharacterized protein | -6.3 |
| IENIEEDFRNGLK | 13 | 1448.53 | A0A669B347 | Uncharacterized protein | -5.8 |
| IGAEEIVDGNVK | 12 | 1243.36 | A0A669B347 | Uncharacterized protein | -6.9 |
| IIDQNRDGIIS | 11 | 1243.37 | A0A669BQ46 | Myosin light chain, phosphorylatable, fast skeletal muscle a | -5.8 |
| IIDQNRDGIISK | 12 | 1371.54 | A0A669BQ46 | Myosin light chain, phosphorylatable, fast skeletal muscle a | -5.2 |
| IITNWDDMEKIW | 12 | 1563.77 | I3IYR2 | Uncharacterized protein | -7.2 |
| IKIIAPPERKY | 11 | 1327.61 | I3IYR2 | Uncharacterized protein | -6.1 |
| INPNSMFDIQVK | 12 | 1405.62 | I3JBN0 | Alpha-1,4 glucan phosphorylase | -6 |
| ISDANAEEMTHVE | 13 | 1445.51 | I3KMQ5 | PDZ domain-containing protein | -6.3 |
| ITNWDDMEKIW | 11 | 1450.61 | I3IYR2 | Uncharacterized protein | -7.8 |
| KDGKIDFDEFLK | 12 | 1454.62 | A0A669E1J4 | Uncharacterized protein | -6.7 |
| KDINIDIPIF | 10 | 1187.38 | A0A669D936 | Uncharacterized protein | -7 |
| KEDKYEEEIKVL | 12 | 1409.54 | A0A669EEE4 | Uncharacterized protein | -5.7 |
| KEIDDLKIK | 9 | 1101.29 | I3JXG6 | Uncharacterized protein | -6.7 |
| KELEEKMVT | 9 | 1106.29 | A0A669BWI2 | Uncharacterized protein | -5.7 |
| KMEGDLNEMEIQ | 12 | 1436.61 | A0A669CK87 | Uncharacterized protein | -4.5 |
| KNSYEEALDHLE | 12 | 1447.5 | A0A669CK87 | Uncharacterized protein | -4.9 |
| KPEEEKQLVK | 10 | 1227.41 | A0A669CD06 | L-lactate dehydrogenase | -5.8 |
| KQEYDEAGPSIVH | 13 | 1472.56 | I3IYR2 | Uncharacterized protein | -6.3 |
| KSADDIKKAF | 10 | 1122.27 | I3KV11 | Uncharacterized protein | -6.2 |
| KTDDGKPFPQ | 10 | 1132.22 | I3KW15 | Fructose-bisphosphate aldolase | -6.5 |
| KTDDGKPFPQYL | 12 | 1408.55 | I3KW15 | Fructose-bisphosphate aldolase | -7.2 |
| KTDKEIDDLKIK | 12 | 1445.66 | I3JXF9 | Uncharacterized protein | -6 |
| KTIDDLEDELY | 11 | 1353.43 | A0A669EEE4 | Uncharacterized protein | -5.5 |
| KTIDDLEDELYA | 12 | 1424.51 | A0A669EEE4 | Uncharacterized protein | -5.2 |
| KTIDDLEDELYAQ | 13 | 1552.63 | A0A669EEE4 | Uncharacterized protein | -5.5 |
| KTIDDLEDEVY | 11 | 1339.4 | I3K0E5 | Uncharacterized protein | -6.1 |
| KVDSELPVDDVFVQ | 14 | 1589.74 | I3KCM4 | Adenylate kinase isoenzyme 1 | -5.3 |
| LAEKDEEMEQIK | 12 | 1462.62 | A0A669CK87 | Uncharacterized protein | -4.4 |
| LAGKSADDIKKAF | 13 | 1363.56 | I3KV10 | Uncharacterized protein | -6.9 |
| LDANLKPIKPM | 11 | 1239.52 | I3J820 | Phosphoglycerate mutase | -6.9 |
| LDDLQAEEDKVN | 12 | 1388.43 | A0A669CK87 | Uncharacterized protein | -6.5 |
| LDDLQAEEDKVNT | 13 | 1489.53 | A0A669CK87 | Uncharacterized protein | -5.9 |
| LDFKSPDDPSRH | 12 | 1413.49 | A0A669C1U1 | 2-phospho-D-glycerate hydro-lyase | -6.6 |
| LDFTENPDELFE | 12 | 1468.51 | A0A669F036 | Collagen type VI alpha 3 chain | -5.6 |
| LDLKDMPFY | 9 | 1141.33 | I3JS34 | Nucleoside diphosphate kinase B | -6.7 |
| LDLKDMPFYA | 10 | 1212.41 | I3JS34 | Nucleoside diphosphate kinase B | -6.3 |
| LEVEPSDTIENVK | 13 | 1472.59 | P62979 | Ubiquitin-40S ribosomal protein S27a | -4.2 |
| LGEQIDNLQRVK | 12 | 1412.59 | A0A669CK87 | Uncharacterized protein | -5.7 |
| LHLDDALR | 8 | 952.06 | A0A669CK87 | Uncharacterized protein | -6.4 |
| LHVDPDNFRVL | 11 | 1324.48 | A0A669EM02 | GLOBIN domain-containing protein | -6.9 |
| LIPNETKTPGLM | 12 | 1313.56 | A0A669CK87 | Uncharacterized protein | -5.7 |
| LPDFDINLK | 9 | 1074.22 | A0A669F3B5 | Uncharacterized protein | -6.4 |
| LQDLVDKLQLKVK | 13 | 1539.85 | A0A669CK87 | Uncharacterized protein | -4.1 |
| LRVFDKEGNGTVM | 13 | 1465.67 | I3K6V3 | Uncharacterized protein | -5.8 |
| LSVDDGKDRVS | 11 | 1190.26 | A0A669F036 | Collagen type VI alpha 3 chain | -7.5 |
| MEDLQDMFIVH | 11 | 1377.58 | A0A669B347 | Uncharacterized protein | -7.3 |
| MFEQSQIQEYK | 11 | 1430.58 | A0A669BQ46 | Myosin light chain, phosphorylatable, fast skeletal muscle a | -5.6 |
| NIDDLTGHLSK | 11 | 1212.3 | A0A669CKH1 | GLOBIN domain-containing protein | -7.2 |
| NIEEDFRDGLK | 11 | 1335.41 | A0A669CHY2 | Alpha-actinin cytoskeletal isoform | -7.3 |
| NLISEIEDGAFSK | 13 | 1422.53 | I3IVI1 | Mimecan | -6.7 |
| NLKGGDDLDPNYVL | 14 | 1532.65 | I3JLI3 | Creatine kinase | -8.3 |
| NSYEEALDHLE | 11 | 1319.33 | A0A669CK87 | Uncharacterized protein | -6 |
| NWDDMEKIW | 9 | 1236.35 | I3IYR2 | Uncharacterized protein | -6.8 |
| NWDDMEKIWH | 10 | 1373.49 | I3IYR2 | Uncharacterized protein | -7 |
| NWDDMEKIWHHTF | 13 | 1758.9 | I3IYR2 | Uncharacterized protein | -8.3 |
| PFDQDDWEHWAK | 12 | 1445.44 | A0A669C1U1 | 2-phospho-D-glycerate hydro-lyase | -7.6 |
| PGPPVLPPNFK | 11 | 1162.37 | I3KF54 | Tryptophanyl-tRNA synthetase | -8.3 |
| PPKFDGGAPVK | 11 | 1112.27 | I3KVU6 | Uncharacterized protein | -7.7 |
| QLHLDDALR | 9 | 1080.19 | A0A669CK87 | Uncharacterized protein | -6.6 |
| RALTDAETKTFLK | 13 | 1493.7 | I3KV10 | Uncharacterized protein | -6.6 |
| RDNLADDLQKLK | 12 | 1428.59 | A0A669DRG9 | Desmin a | -6.4 |
| REFDDLPLH | 9 | 1141.23 | A0A669BZJ2 | Calcium-transporting ATPase | -8.5 |
| RIGEDFIRD | 9 | 1120.21 | I3JBN0 | Alpha-1,4 glucan phosphorylase | -7.1 |
| SDNFDDYMK | 9 | 1134.17 | I3J359 | FABP domain-containing protein | -6.6 |
| SDPRDIDEDAILR | 13 | 1358.41 | A0A669DLY7 | Uncharacterized protein | -6.4 |
| SGDLEEELKNVT | 12 | 1333.39 | I3K0E5 | Uncharacterized protein | -5.5 |
| SGPPVPGPIGPM | 12 | 1105.3 | I3JMB4 | Uncharacterized protein | -6.3 |
| SLSSKDKTVVK | 11 | 1191.37 | A0A669CKH1 | GLOBIN domain-containing protein | -5.3 |
| SPDDPSRH | 8 | 909.9 | A0A669C1U1 | 2-phospho-D-glycerate hydro-lyase | -5.8 |
| SPKQPDLFKEIVN | 13 | 1514.72 | I3JBN0 | Alpha-1,4 glucan phosphorylase | -5.5 |
| SQKEDKYEEEIKVL | 14 | 1396.5 | A0A669EEE4 | Uncharacterized protein | -5.7 |
| SVDDEFPDLTK | 11 | 1265.32 | I3JLI3 | Creatine kinase | -5.8 |
| SVDDYAEMLR | 10 | 1198.3 | A0A669CLU6 | Phosphoglucomutase 1 | -6.4 |
| TDDGKPFPQY | 10 | 1167.22 | I3KW15 | Fructose-bisphosphate aldolase | -7.4 |
| TDKEIDDLKIK | 11 | 1317.48 | I3JXF9 | Uncharacterized protein | -5.1 |
| TEEDKKNLVRL | 11 | 1344.51 | A0A669CK87 | Uncharacterized protein | -5.3 |
| TEQEKTPLQ | 9 | 1073.15 | A0A669BZJ2 | Calcium-transporting ATPase | -6.3 |
| THPKFEEILTR | 11 | 1370.55 | I3JLI3 | Creatine kinase | -7.7 |
| TIIDQNRDGIIS | 12 | 1344.47 | A0A669BQ46 | Myosin light chain, phosphorylatable, fast skeletal muscle a | -7.4 |
| TIIDQNRDGIISK | 13 | 1472.64 | A0A669BQ46 | Myosin light chain, phosphorylatable, fast skeletal muscle a | -6 |
| TLDDLQAEEDKVNT | 14 | 1590.64 | A0A669CK87 | Uncharacterized protein | -5.4 |
| TNPYDYPMIS | 10 | 1200.31 | A0A669CK87 | Uncharacterized protein | -7.5 |
| TNWDDMEKIW | 10 | 1337.45 | I3IYR2 | Uncharacterized protein | -8.2 |
| TPDQIEDFK | 9 | 1092.15 | I3K6V3 | Uncharacterized protein | -7.4 |
| TPEQIEDFKDAFQ | 13 | 1439.52 | I3JSP5 | Uncharacterized protein | -7.6 |
| TSERLEDEEEIN | 12 | 1463.45 | A0A669CK87 | Uncharacterized protein | -5.5 |
| VDDEFPDLTK | 10 | 1178.24 | I3JLI3 | Creatine kinase | -7.4 |
| VDFEKTKEDLAK | 12 | 1422.57 | A0A669BWI2 | Uncharacterized protein | -5.5 |
| VFDISNADRLG | 11 | 1206.3 | I3JLI3 | Creatine kinase | -8.6 |
| VFEWENFAK | 9 | 1169.28 | I3KL67 | Phosphoglycerate kinase | -8.2 |
| VFPGIPDHLDA | 11 | 1180.3 | A0A669C9W7 | Uncharacterized protein | -7.9 |
| VGDKVPADIRIVS | 13 | 1368.57 | A0A669BZJ2 | Calcium-transporting ATPase | -7.3 |
| VIEPLDIGDDKVR | 13 | 1468.65 | A0A669BIT2 | Collagen type VI alpha 3 chain | -9 |
| VINDNFGIIEGLM | 13 | 1434.65 | A0A669BWU8 | Glyceraldehyde-3-phosphate dehydrogenase | -7 |
| VKEDDIHPM | 9 | 1083.21 | A0A669EC89 | Uncharacterized protein | -7.3 |
| VNEEDHLRVI | 10 | 1223.33 | I3JLI3 | Creatine kinase | -6.3 |
| VNEEDHLRVIS | 11 | 1310.41 | I3JLI3 | Creatine kinase | -5.7 |
| VWEGQNIVKL | 10 | 1185.37 | I3JS34 | Nucleoside diphosphate kinase B | -7 |
| WDDMEKIW | 8 | 1122.24 | I3IYR2 | Uncharacterized protein | -7 |
| YDDIKKVVK | 9 | 1107.29 | A0A669BWU8 | Glyceraldehyde-3-phosphate dehydrogenase | -5.7 |
| YFPGDFTPEVH | 11 | 1308.39 | A0A669CKH1 | GLOBIN domain-containing protein | -7.2 |
| YSQKEDKYEEEIK | 13 | 1446.51 | A0A669EEE4 | Uncharacterized protein | -5.3 |
