# Supplementary material for: Identification and virtual screening of novel salty peptides from hydrolysate of tilapia by-product by batch molecular docking
Source: Front Nutr. 2024 Jan 8;10:1343209. doi: 10.3389/fnut.2023.1343209 (PMC10800615; doi:10.3389/fnut.2023.1343209)
Supplement: Supplementary file 2 [file Table_2.docx]

**Supplementary Table 2** Taste attributes and threshold values of 16 synthetic peptide water solutions and their docking energy with TRPV1.

| Peptide | Length | Mass (Da) | Docking energy  (kcal/mol) | Taste Description | Threshold  Value (mmol/L) |
| --- | --- | --- | --- | --- | --- |
| DFKSPDDPSRH | 11 | 1300.33 | -8.0 | Salty, sour, slight astringent | 0.261±0.013 |
| DPFDQDDWEAWTK | 13 | 1652.67 | -8.1 | Salty, slight sour, astringent | 0.283±0.032 |
| FIYDVLEDPVE | 11 | 1338.46 | -8.2 | Salty, sour, astringent | 0.333±0.045 |
| FPGDFTPEVH | 10 | 1145.22 | -9 | Salty, sour, slight astringent | 0.307±0.025 |
| FPGIPDHL | 8 | 895.01 | -8.3 | Salty, sour, slight astringent | 0.295±0.038 |
| FSADQIEDYR | 10 | 1243.28 | -8.5 | Salty, slight sour, astringent | 0.312±0.011 |
| GEIDEFLPAPR | 11 | 1243.36 | -8.6 | Salty, slight sour, astringent | 0.332±0.035 |
| HLDDALR | 7 | 838.91 | -8.4 | Salty, sour, slight astringent | 0.278±0.031 |
| NLKGGDDLDPNYVL | 14 | 1532.65 | -8.3 | Salty, slight astringent | 0.291±0.039 |
| NWDDMEKIWHHTF | 13 | 1758.91 | -8.3 | Salty, slight astringent | 0.294±0.023 |
| PGPPVLPPNFK | 11 | 1162.38 | -8.3 | Salty, slight sour, slight astringent | 0.379±0.045 |
| REFDDLPLH | 9 | 1141.23 | -8.5 | Salty, sour, astringent | 0.343±0.011 |
| TNWDDMEKIW | 10 | 1337.46 | -8.2 | Salty, slight astringent | 0.290±0.010 |
| VFDISNADRLG | 11 | 1206.31 | -8.6 | Salty, slight sour, astringent | 0.322±0.012 |
| VFEWENFAK | 9 | 1169.28 | -8.2 | Salty, slight astringent | 0.328±0.011 |
| VIEPLDIGDDKVR | 13 | 1468.65 | -9 | Salty, slight sour, slight astringent | 0.256±0.016 |
