# Supplementary figures and images for: Identification and virtual screening of novel salty peptides from hydrolysate of tilapia by-product by batch molecular docking
Source: Front Nutr. 2024 Jan 8;10:1343209. doi: 10.3389/fnut.2023.1343209 (PMC10800615; doi:10.3389/fnut.2023.1343209)

**
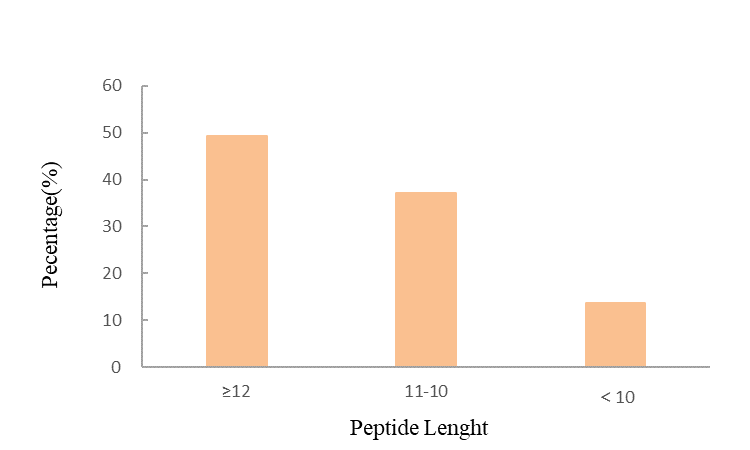
**

**Fig. S1** Peptide length distribution of retained peptides.

Supplement: Supplementary file 3 [file Data_Sheet_1.docx]
